# Equimetrix Device: Criteria Based Validation and Reliability Analysis of the Center of Mass and Base of Support of a Human Postural Assessment System

**DOI:** 10.3390/s21020374

**Published:** 2021-01-07

**Authors:** Pedro Fonseca, Manoela Sousa, Ricardo Sebastião, Márcio Goethel, Pierre Barralon, Igone Idigoras, Filipa Sousa, Leandro Machado, João Paulo Vilas-Boas

**Affiliations:** 1 Porto Biomechanics Laboratory, University of Porto (LABIOMEP-UP), 4200-450 Porto, Portugal; manoelavsousa@fade.up.pt (M.S.); rsebastiao@fade.up.pt (R.S.); gbiomech@gmail.com (M.G.); filipas@fade.up.pt (F.S.); lmachado@fade.up.pt (L.M.); jpvb@fade.up.pt (J.P.V.-B.); 2 Faculty of Sport, University of Porto (FADEUP), 4200-450 Porto, Portugal; 3 TECNALIA, Basque Research and Technology Alliance (BRTA), Parque Científico y Tecnológico de Gipuzkoa, Mikeletegi Pasealekua 2, E-20009 Donostia-San Sebastián, Spain; pierre.barralon@tecnalia.com (P.B.); igone.idigoras@tecnalia.com (I.I.)

**Keywords:** center of mass, base of support, balance, stability, human postural control, validation study

## Abstract

Human postural control is a fundamental ability for static and dynamic tasks, especially in hiper- and hipo-functional populations, such as the elderly. The Equimetrix is a clinical device developed to assess both the base of support (BoS) and the center of mass (CoM) dynamics, thus allowing their use as new evaluation and training tools. This study aims to perform a criteria based validation of Equimetrix by comparing the BoS and CoM data with gold-standard equipment. A motion capture system, force platform, and pressure mat were used to calculate the CoM, center of pressure (CoP) and BoS during bipedal, unipedal, feet together and full tandem stances. Results demonstrate an excellent reliability of Equimetrix in terms of spatial accuracy of the CoM, although over-estimating the CoM height. Differences were found when comparing Mean velocity Path with the CoM, but not with the CoP, indicating a lower reliability in time-based parameters. The Equimetrix presents a tendency to overestimate the BoS, with mixed reliability values, which may be related to the different size of sensing elements between the Equimetrix and the pressure sensing mat. These are encouraging results that should be further explored during dynamic tasks.

## 1. Introduction

The ability to maintain balance is a necessary requirement for static and dynamic postural control in humans and is considered a key element in daily living and sports activities [1,2]. This is a complex process regulated by the nervous, visual, vestibular, and motor systems, which are responsible for the transmission of the necessary information to the somatosensory system in order to trigger an adequate motor response [1,3]. Evaluating balance is important in different populations, but particularly for athletes, as hyper functional persons, and for patients and elderly, as hypo functional subjects prone to falls and injury.

The quality of life of ageing citizens has been a topic of research and concern for scientists and healthcare companies alike. This is supported by the staggering number of senior citizens that reported at least one fall per year (65 years old: 30%; 80 years old: 50%) [4]. Although not all causes of fall can be solved with training, the application of physical exercises, including proprioceptive and particularly balance training, has shown evidence of a positive effect in fall prevention [5].

To keep a proper postural control during quasi-static erect position, the body’s Center of Mass (CoM) vertical projection must be within the lower limb’s defined Base of Support (BoS) [6,7]. Due to the principle of free energy [8], the systems responsible for the postural regulation and balance will try to match vertically the CoM with the Center of Pressure (CoP), which represents the point of application of the ground reaction forces. The CoP migration is the most commonly used method to assess human balance [9,10]. Although the CoM and CoP may present similar variations in a semi-static erect position [11], they represent different concepts [12], with the first being related to the body’s sway and the second to its neuromuscular response [9].

To evaluate human balance, the literature has reported several objective methods such as the use of force platforms [9,13,14], electromyography [3,15], as well as more clinical test as the Star Excursion Balance Test [16], the Y Balance Test [17], and even the more interactive means provided by Exergames [18]. Although several equipment, methods, and systems are available for balance assessment in laboratory context, they are usually complex and expensive. Cost-effective solutions, that can be used as a first stage of evaluation, or even as a complement [19,20], could become a high dissemination method. With affordable balance assessment solutions, the installation at the patient’s house could even be made possible [21]. Therefore, the easy access to affordable and reliable systems of balance assessment and training could prove to be a decisive instrument to reduce elderly, pathological, or any other populations’ exposure to risk.

The Wii Balance Board is a good example of an affordable, portable, and easy to use device for balance training. Initially presented as a peripheral for videogames, it has gained some traction as an alternative to force platforms by using it along with custom software [22,23,24]. A review [25] on the use of this system has shown that although being a valid instrument, it has shortcoming that should be considered when using it. Other approaches have been focused on the use of the ubiquitous smartphones, where its accelerometer and gyroscope information can be used to estimate the CoM movement in stroke patients [26]. The willingness to use gaming hardware and technologies by clinical professional can be due to the lack of custom and well-adapted alternatives to their needs [19]. While the Wii Balance Board can be seen as an alternative to regular force platforms, alternatives to motion capture-based postural assessment is also available. The use of RGB-D cameras, such as the Microsoft Kinect, has been explored for analysis of gait [27,28,29], postural control [30,31,32], and posture tracking [33], and is yet another instance of the use of mass-produced gaming hardware applied to science. The Kinect can be used as a reliable alternative to laboratory-based motion capture systems [34,35], while still allowing the measurement of both posture and movement. However, and despite the technology used for implementation, both the Wii Balance Board and the RGB-D cameras, perform their balance assessment based either on CoP or CoM migration, not enabling a comprehensive analysis of how these parameters relate with the BoS. 

Rather than an individual analysis of either the CoM or CoP, the joint analysis of these parameters, or even the addition of BoS, has been proposed [36,37]. This allows the use of metrics such as the extrapolated CoM (XCoM), Dynamic Stability Margin (DSN), Margin of stability (MoS), XCoM-CoP Distance, and CoM-CoP angle, which have been demonstrated to be relevant and reliable [38]. As pointed before, the access to such comprehensive measurements would require dedicated facilities equipped with, at least, motion capture and force platform systems, which are not affordable and easy to maintain for clinicians, physiotherapists, or occupational therapists. Table 1 presents a comparison between current technologies that enables the measurement of human CoP, CoM or BoS, but also those suitable for the simultaneous measurement of both CoM and BoS. 

As far as we know, currently there is no commercial solution that can measure and combine both CoM and BoS information during static and dynamic standing as well as for stepping responses. Equimetrix is the first portable clinical device measuring the functional state of balance by combining two highly relevant human motion descriptors: center of mass and foot related information (BoS). By functional assessment, it covers upright posture but more interestingly also dynamic tasks such as body weight shift, step response, and reaching a high shelf. The Equimetrix system intends to be an affordable, portable, and easy to use platform to measure balance control that can be reliably used and deployed in several contexts. The use of such an equipment could promote easier assessment of the patients’ balance control as well as providing senior citizens with an easy to use tool to include in their training and evaluations routines.

The purpose of this study is to evaluate how the Equimetrix device performs in terms of determination of different bases of support, and if it can be reliably used as a postural control measuring system in humans, by comparing its measurements to CoM, CoP, and BoS data retrieved from gold standard system and using common stabilogram and statokinesigram parameters as validation criteria. We hypothesize that Equimetrix will present differences when compared to the gold standard devices, especially in time-dependent parameters. We also expect to find these differences when comparing to the CoP data, as the Equimetrix device is based on CoM calculations.

## 2. Materials and Methods

### 2.1. Participants

A convenience sample was recruited from the local academic population, comprising 8 male (age: 30.8 ± 8.5 years; height: 178.4 ± 6.6 m; mass: 81.8 ± 10.8 kg) and 12 female (age: 33.8 ± 9.4 years; height: 161.9 ± 5.5 m; mass: 63.2 ± 6.8 kg) volunteers. Only subjects with no muscular-skeleton lesions in the 6 months before data collection, no neurological or motor disorders and no visual or hearing impairments, were included in the study.

All participants gave their informed consent for inclusion before they participated in the study. The study was conducted in accordance with the Declaration of Helsinki, and the protocol was approved by the Faculty of Sports of the University of Porto Ethics Committee with the number CEFAD 26/2020.

### 2.2. The Equimetrix Measuring System

During the last 30 years, instrumental tools have been suggested to evaluate quantitatively postural balance parameters. Measure stability directly is impossible. It is not a magnitude, but merely an aptitude, enabling the body to return close to its position of equilibrium whenever strays from it. However, stability has characteristics that indeed can be measured as referred in the introduction.

Force and stabilometric platforms (Ground Reaction Force, CoP) are currently the most used devices. CoP measurement alone reflects balance skills when the user/patient is in static condition. However, CoP measurement alone does not reflect postural control skills when users/patients are performing dynamic activities such as body weight shift, reaching object, tying shoes, and walking. 

For such activities, the joint measurement of CoM and CoP (or base of support) is much more relevant. Scientific evidence supports the paradigm that a joint analysis of CoM-CoP or CoM-Foot placement reflect human functional stability [39,40].

A standard motion capture system combined with a baropodometric device are able to estimate functional stability by combining user’s CoM and BoS. However, there is a need for an integrated, portable, and low-cost technology that can perform similar measurements and estimation of functional stability that can largely be deployed among (social) healthcare professionals. Equimetrix presents this integration.

**Figure 1:**
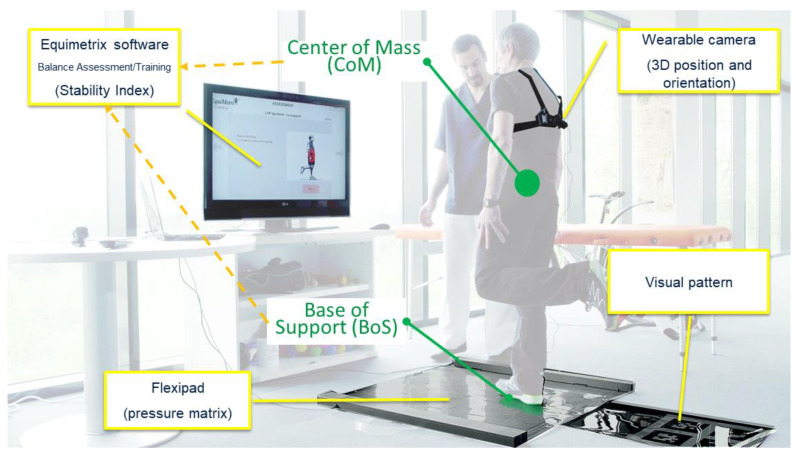
Equimetrix main components: sensing mat, wearable camera, and reference visual pattern.

The Equimetrix system (Figure 1) comprises a sensing mat and a portable optical sensor to allow the simultaneous measurement of the BoS and the CoM positions, respectively. The sensing mat consists in six flexible touch pads (45.0 × 30.0 cm), comprising 216 sensors (2.5 × 2.5 cm) each. These pads are seamless connected to provide an overall mat area of 90 × 90 cm with a total of 1296 touch sensors.

**Figure 2:**
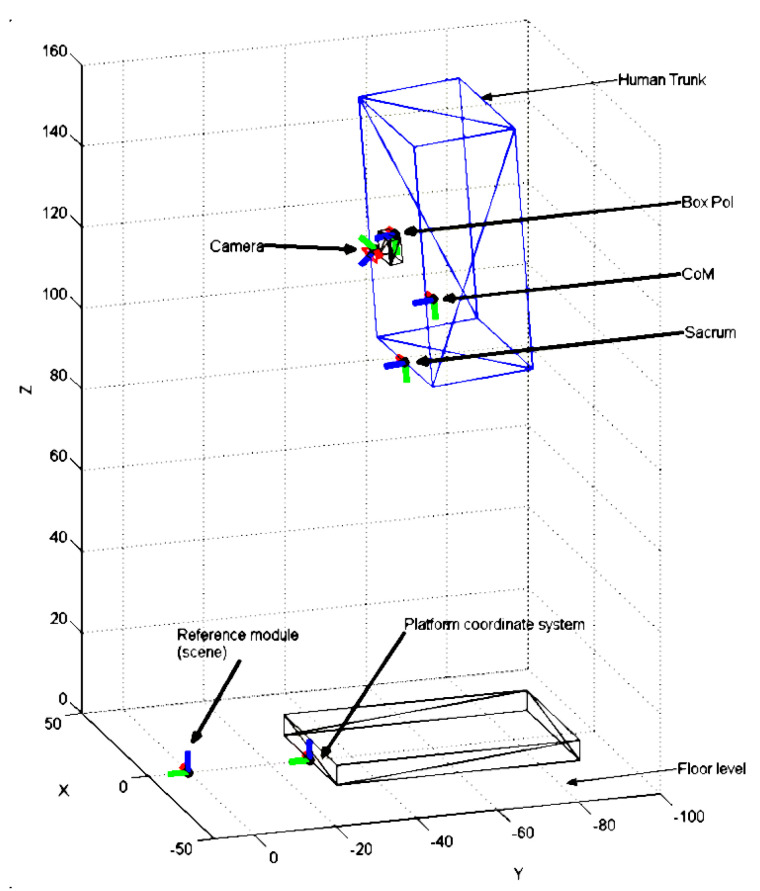
Illustration of the various frame coordinates used in Equimetrix to estimate CoM position with respect to a reference frame (“scene”). The blue box represents the subject’s trunk, while the Box PoI stands for “Point of Interest” and corresponds to a particular location on the casing holding the camera.

An optical sensor is built around a webcam secured on a harness that can be positioned on a person’s back and a reference optical pattern placed on the ground, posterior to the sensing mat and responsible for estimating the subject’s CoM. This is calculated taking in account that the trunk comprises 50% of the total body mass [41]. The working principle is that the camera will be able to detect the optical pattern, thus creating a reference to which the camera’s movement will be estimated, based on homographic transformation properties (Figure 2).

The 3D position of the CoM in the scene reference frame (CoMscene) is computed, according to Equation (1), as follows:

(1) CoMscene=(Tcamscene×TboxCoM×Tsacbox×TCoMsac)×CoMCoM

from which most transformation matrices are known:

Tcamscene is a 4 × 4 transformation matrix that expresses the 3D translation and rotation needed to convert 3D points expressed in the “camera frame” to the “scene frame”.

TboxCoM: matrix transforming points expressed in the “box” (sensor casing) frame to the camera frame. This matrix depends on the mechanical design of the wearable sensor.

Tsacbox: matrix transforming points expressed in the “sacrum” of the subject frame to the box frame. Even though the location of the wearable sensor was different for each user, this transformation matrix was constant for all users by using an average distance (sensor–sacrum) determined in previous experiments performed in Tecnalia’s motion laboratory.

TCoMsac: matrix transforming points expressed in the “CoM” frame to the sacrum frame. this transformation matrix was constant for all users and using an average transformation determined in previous experiments performed in Tecnalia’s motion laboratory.

**Figure 3:**
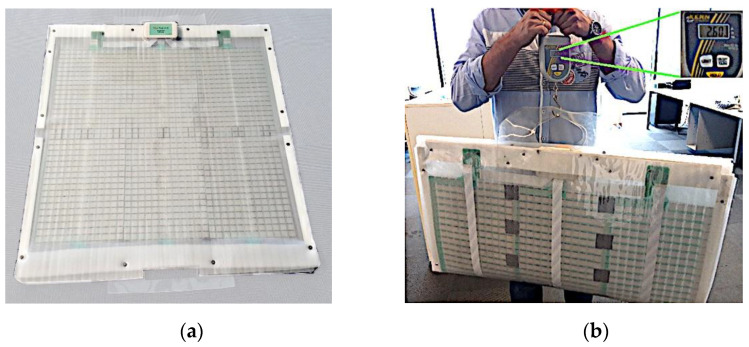
(**a**) Most recent version of the Equimetrix sensing mat and (**b**) its weight measurement (2.6 kg) in the folded transport configuration.

Thanks to its concise design, Equimetrix sensing mat weighs only 2.6 kg and can be folded in two parts for easy transportation (Figure 3). Both these features, along with the large sensing area available, present an increased advantage when compared to traditional stability measurement devices, or even to other custom made solutions designed for portability and low cost [42]. 

**Figure 4:**
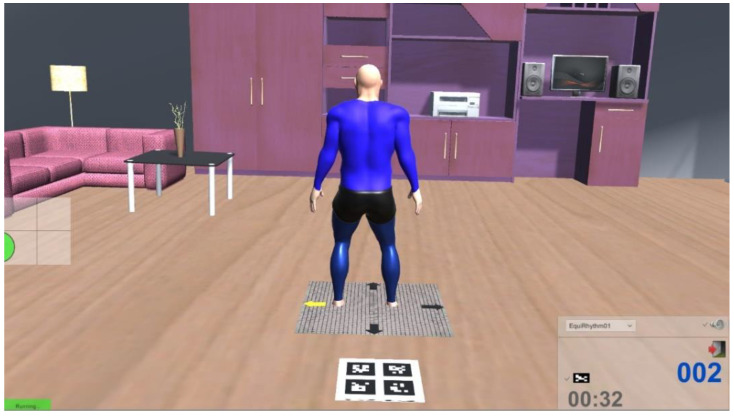
Illustration of the training game “Dancing”, where at the stage the user is asked to perform directional weight transfers according to the indications given by the yellow arrow.

The comprehensiveness of the Equimetrix solution is even improved by the inclusion of a graphical interface supporting system configuration, balance assessment programs (e.g., Short Physical Performance Battery, functional tasks, etc.), and balance training modules based on serious games. An example of a game where the subject’s weight transfers can be used to interact with an immersive virtual environment is illustrated on Figure 4.

### 2.3. Experimental Setup and Data Collection Procedures

#### 2.3.1. Center of Mass and Center of Pressure

The Equimetrix sensory mat was positioned on a Bertec force platform with a 60 × 90 cm area (Bertec Inc., Columbus, OH, USA) so that the center of the Equimetrix mat was aligned with the center of the force platform. The forces, moments and center of pressure data were acquired at 2000 Hz and in synchrony with a kinematic system.

A 12-camera motion capture system (Qualisys AB, Göteborg, Sweden), operating at a sampling frequency of 200 Hz, was calibrated so that a working volume of approximately 1.0 × 1.0 × 2.0 m (2.0 m^3^) was located over the Equimetrix mat. An additional volume of approximately 1.0 × 1.0 × 0.25 m (0.25 m^3^) was calibrated over the Equimetrix’s optical pattern sheet. Only calibrations with an error below 0.5 mm were accepted.

**Figure 5:**
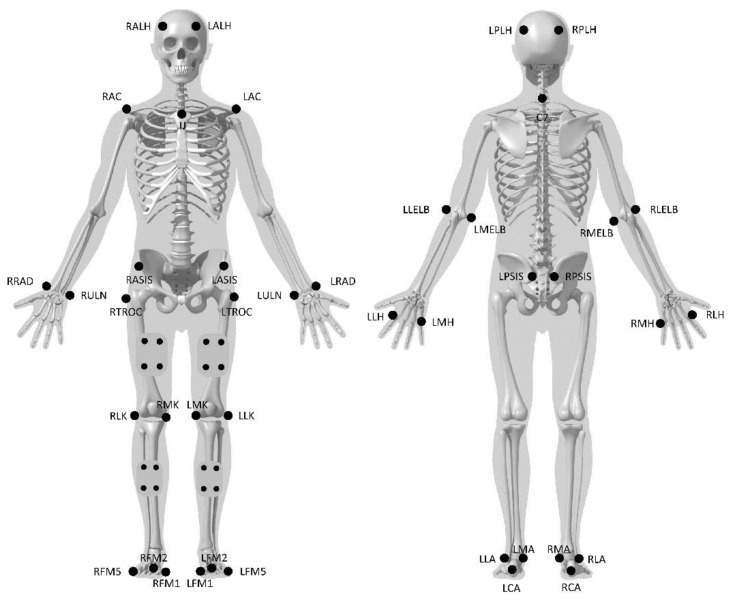
Anatomical position of the retroreflective markers. RALH/LALH: right/left anterior left head; RPLH/LPLH: right/left posterior lateral head; RAC/LAC: right/left acromion; C7: seventh cervical vertebra; IJ: incisura jugularis; RLELB/LLELB: right/left lateral elbow; RMELB/LMELB: right/left medial elbow; RRAD/LRAD: right/left radium; RULN, LULN: right/left ulna; RLH/LLH: right/left lateral hand; RMH/LMH: right/left medial hand; RASIS/LASIS: right/left anterior superior iliac crest; RPSIS, LPSIS: right/left posterior superior iliac spine; RTROC/LTROC: right/left great trochanter; RLK/LLK: right/left lateral knee; RMK/LMK: right/left medial knee; RLA/LLA: right/left lateral ankle; RMA/LMA: right/left medial ankle; RCA/LCA: right/left calcaneus; RFM1/LFM1: right/left first metatarsal bone; RFM2/LFM2: right/left second metatarsal bone; RFM5/LFM5: right/left fifth metatarsal bone.

Retroreflective markers were placed on the subject with a full-body configuration in order to model each segment, as illustrated in Figure 5. Markers were placed on the anterior and posterior aspect of the head at the level of the right and left temple; on the right and left acromion; on the incisura jugularis; on the seventh cervical vertebra; on the lateral and medial epicondyles of the right and left humerus; on the right and left radial and ulnar heads; on the lateral aspect of the second metacarpal bone and on the medial aspect of the fifth metacarpal bone of each hand; on the right and left anterior and posterior iliac crests; right and left great trochanter; lateral and medial epicondyles of the right and left femur; lateral and medial malleoli of both limbs; and medial aspect of the first metatarsal head, lateral aspect of the fifth metatarsal head and on top of the second metatarsal head of both feet.

The Equimetrix camera was strapped on the subject and its angle adjusted according to the manufacturer, so that the optical pattern was in the field of view.

The subject was then positioned barefoot on an A3 paper sheet (29.7 × 42.0 cm) placed at the center of the sensory mat and the feet contour was drawn for each foot position [43]. In order to assess the validity of the Equimetrix device to measure postural control and base of support with several standard feet positioning, the following positions were considered [10]: bipedal with feet apart 10 cm at the malleoli (BI), feet together (FT), unilateral support over the dominant lower limb (UNI), and full tandem (fullT) with the dominant foot forward.

Prior to data collection, and after placement of the motion capture markers on the subject, a familiarization with the testing positions was performed on the Equimetrix sensing mat. Each subject was allowed to test the selected feet positions until they felt comfortable and confident and they were able to perform them for the duration of the data acquisition trial.

Data collection comprised a 60-second trial for each foot position, performed in a randomized order by each subject on a static position, with the arms standing parallel to the body, while gazing a fixed point at the eye level and at a distance of 2 m. The subject was instructed not to speak, move the head, or perform any adjustment movement during the period of data collection. If the subject expressed any discomfort during a given position, the test would be stopped, the subject allowed to adjust to once again be comfortable, and then data collection would resume.

#### 2.3.2. Base of Support

Each foot position sheet was randomly placed over a pressure sensitive mat (Walkway, Tekscan, Boston, MA, USA) and the subject was instructed to step on it and adjust the feet so they would be in agreement with the previously drawn layout. Once this was accomplished, a 10-second recording with the subject standing still with the arms parallel to the body was performed with the Tekscan software (Tekscan, Boston, MA, USA) at a sampling frequency of 100 Hz, thus collecting a pressure map of the feet.

**Figure 6:**
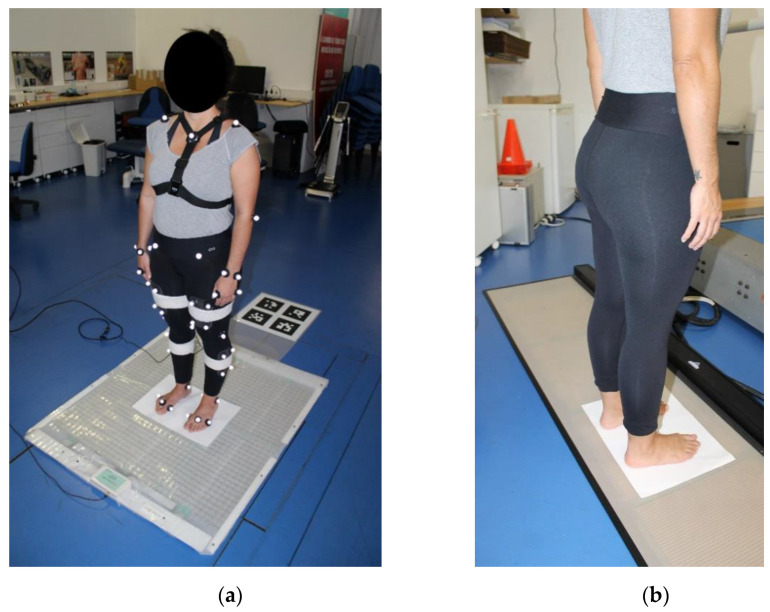
Representation of the center of mass experimental setup with a participant (**a**) standing on the Equimetrix mat while wearing the camera and retroreflective markers and (**b**) on the podobarometric pressure platform during the base of support measurement.

A representation of the experimental setup for the measurement of the base of support, as well of the center of mass and center of pressure is depicted in Figure 6.

#### 2.3.3. System Synchronization

The kinematic and force data were electronically synchronized, with the force platform data being acquired with a dedicated analog-to-digital converter (National Instruments, Austin, TX, USA) and the Qualisys Track Manager software (Qualisys AB, Göteborg, Sweden).

In the same manner, the Equimetrix sensory mat and its camera were also electronically synchronized with each other, and data was collected with its own dedicated software.

Since the Equimetrix device does not possess an electronic synchronization input or output, allowing synchronization with third-party equipment, a method of mechanical synchronization was devised. This consisted of a researcher performing a step on the sensory mat, which would be recorded simultaneously by the sensory mat as a momentary increase in the base of support and by the force platform as an increase of the vertical force. This was performed at the beginning and ending of each trial.

### 2.4. Data Processing and Analysis

#### 2.4.1. Center of Mass and Center of Pressure Analysis

The kinematic data was processed in the Qualisys Track Manager software and comprised the marker identification, removal of artefacts, and gap-filling trajectories.

The kinetic and kinematic data was then exported to Visual3D (C-Motion Inc., Germantown, MD, USA) where a full body reconstruction with 6 degrees of freedom segments was performed, thus allowing the calculation of the subject’s kinematic center of mass (K_CoM). The force platform center of pressure (F_CoP) was also calculated with the Visual3D built-in routines. A common global coordinate system was established for all measuring systems.

The K_CoM, F_CoP, as well as the Equimetrix center of mass (*E*_*CoM*) data were then further processed with a custom Matlab R2019a (Mathworks, Natick, MA, USA) routine which performed the data synchronization, time normalization, smoothing, and extraction of the metrics used in the statistical analysis. Data synchronization was performed by selecting the range of data between the mechanical synchronization events. These data points were then time-normalized so that the first point would correspond to 0% and the last to 100% of the task. A smoothing filter was applied to remove unwanted noise and transient effects using a moving average with a window of 2% normalized time. The resulting anterior-posterior (AP) and medial-lateral (ML) signals had their mean removed in order to be centerd around zero. The mean of the K_CoM and *E*_*CoM* vertical component was not removed nor centerd around zero since their absolute value was of importance for the subsequent analysis, but also because the floor acted as a common reference point.

Posturography data can be analyzed as one-dimensional through the stabilogram, which presents the center of pressure migration as independent time-series on the AP and ML directions, or as a two-dimensional time-series known as the statokinesigram [44]. A selection of three one dimension (1D) and five two dimension (2D) parameters [45] were calculated for the K_CoM, F_CoP and *E*_*CoM*. The 1D parameters included the center of pressure mean path velocity, excursion range, and root mean square value. These parameters were calculated for the AP and ML components, but also for the vertical component of K_CoM and *E*_*CoM*. The 2D parameters included the mean center of pressure distance, center of pressure length, 95% prediction ellipse and its large and short axis.

#### 2.4.2. Center of Mass Height Correction

The height of the CoM is a fundamental element in the model of the inverted pendulum. Therefore, a preliminary analysis was performed to verify if the vertical component of the K_CoM and *E*_*CoM* were identical and if the sway angle measured by both systems provided similar results.

#### 2.4.3. Base of Support Analysis

Pressure data recorded with the force platforms and the BoS recorded with the Equimetrix mat were processed and analyzed in a custom Matlab R2019a (Mathworks, Natick, MA, USA) routine. The BoS was defined as a polygon delimited by the outer-edges of the pressure data for each foot position, and the AP and ML lengths of the BoS, as well as the corresponding area were calculated.

### 2.5. Statistical Analysis

Data normality was assessed through the Shapiro–Wilk test, for each variable and regarding feet position. The preliminary analysis of the center of mass height correction was performed with an independent samples t-test for normal parameters and a Mann–Whitney U-test for non-normal data, followed by a linear regression with the vertical component of the *E*_*CoM* as a predictor of the vertical component on the K_CoM.

To verify differences between K_CoM, F_CoP and *E*_*CoM* on all 4 feet positions a MANOVA analysis was used, for the 1D parametric variables, followed by multiple comparisons *t*-tests with Bonferroni adjustment (*p* < 0.017). For the 1D and 2D non-parametric variables, a Kruskall–Wallis *H* test, followed by Mann–Whitney multiple comparisons with Bonferroni adjustment (*p* < 0.017), was used. To analyze de BoS normal and non-parametric variables, independent samples *t*-tests and Mann–Whitney *U* tests were employed, respectively, to verify the differences between the Equimetrix and the pressure mat. 

The effect sizes and observed power were calculated using G*Power v. 3.1.9.2 [46] and interpreted according to Cohen [47] and Sawiloswsky [48] as very small (0.01), small (0.20), medium (0.50), large (0.80), very large (1.20), and huge (2.0). The reliability of the Equimetrix system was assessed with a Two-Way Mixed Model (consistency) Intraclass Correlation Coefficient (ICC). The values of ICC were interpreted according to Youdas, Carey and Garrett [49] as poor (≤0.69), fair (0.70–0.79), good (0.80–0.89), and as excellent (0.80–0.89). Correlations were calculated with the Pearson and Spearman correlation coefficients, for parametric and non-parametric data, respectively, and interpreted according to Portney and Watkins [50] as little or none (0.00–0.24), fair (0.25–0.49), moderate (0.50–0.74), or good (≥0.75). All statistical procedures were performed using SPSS Statistics Version 25.0 (IBM Corporation, Armonk, NY, USA) with a statistically significance defined at *p* < 0.05. 

Sample size for correlation tests was determined by an a priori analysis conducted with G*Power v. 3.1.9.2 and a pilot study with 5 subjects and two feet positions (FT and UNI). Four variables of interest were considered: Range AP and ML (1D variables), Ellipse Area (2D variables), and Average Area (BoS variable). With the minimum lower bound of the 95% confidence interval of the statistically significant correlations (*r* = 0.91, 95% CI: 0.54–1.00, *p* < 0.01) and an α = 0.05, a power of 0.8 was achieved with 19 subjects. 

Results are presented as mean ± standard deviation and median (inter-quartile range) for parametric and non-parametric data, respectively.

## 3. Results

### 3.1. Center of Mass Height Correction

The *E*_*CoM* presents consistently higher values of CoM height in all feet positions (BI: *U* = 4.00, *p <* 0.01; FT: *U =* 3.00, *p* < 0.01; fullT: *t*(32) = −8.73, *p <* 0.0.1; UNI: *t*(30) = −9.67, *p* < 0.01), representing a significant difference between systems. However, the Sway Angle and Sway RMS values do not differ between systems, with the exception of the ML component on the BI position (Sway Angle Range: *U* = 101.00, *p* < 0.01; Sway Angle RMS: *U* = 122.00, *p* = 0.04). This means the CoM sway is identically measured by both systems, albeit the higher *E*_*CoM* value, which may lead to an overestimation of the AP and ML displacement.

Using the K_CoM vertical component as reference, a linear regression equation was calculated to determinate a correction factor. The following Equation (2) was obtained with an adjusted R^2^ = 0.89; *F*(1, 69) = 568.617; *p* < 0.01:

(2) $$E\_CoM_{adjusted}=\;17.36+0.64\times E\_CoM$$

Since the corrected *E*_*CoM* predictors are significant (β = 0.640; *p* < 0.01), the AP and ML components of *E*_*CoM* were recalculated, using the new height ($E\_CoM_{adjusted}$) and the sway angle previously calculated. A summary of the original and corrected CoM height, as well as the sway angle measured with both systems can be found in Table 2.

The underlying statistical results, its observed power and effect size can be consulted in Table 3.

### 3.2. Center of Mass and Center of Pressure

No differences were found in the 1D parameters between the *E*_*CoM* and F_CoP, for any feet position, with the exception of the Mean Path Velocity during the BI position and the ML component of the Mean Path Velocity in the fullT position.

The RMS value of the *E*_*CoM* and K_CoM did not show any differences, in any component, throughout any feet position. Excellent reliability and correlation were also found, but with a decreasing effect size in more unstable positions such as fullT and UNI.

The range of migration is identical between systems in the AP and ML components, with the exception of the ML component during BI stance. The AP range presented reliabilities from good to excellent and correlation between moderate and good. The ML range showed reliability results from poor to excellent and correlations from moderate to excellent, although in some cases significant correlations were not found. The vertical range of migration is different between systems, and is associated with a fair to poor reliability, and without any significant correlation.

The Mean Path Velocity is different between the Equimetrix and the K_CoM measuring system in all their components and positions, with poor to fair reliabilities and small correlations in a few positions. When comparing with the F_CoP, only in BI and in the ML component of the fullT position differences were found with the *E*_*CoM* Path Velocity. Although associated to a very poor reliability and no correlation, there is a large effect size.

The results of the 1D parameters comparison are presented in Table 4, while the corresponding statistical results, observed power and effect size can be consulted in Table 5.

The 2D parameters results, summarized in Table 6, do not show differences between the *E*_*CoM* and the K_CoM or the F_CoP in terms of Mean Distance, Ellipse Area, Ellipse Long Axis, and Ellipse Short Axis. These results are associated to excellent reliability and correlation, albeit with a small effect size. This is supported by the corresponding statistical results, observed power and effect size available in Table 7. The *E*_*CoM* migration length presents differences when compared with the K_CoM in all feet positions, and with the F_CoP in the BI and UNI positions.

When comparing the *E*_*CoM* with the other measuring methods, there is an excellent reliability and good correlation in all parameters and feet positions. The only exceptions occur with the already mentioned differences and with the BI position in general. 

### 3.3. Base of Support

The Equimetrix system shows a non-significant tendency to overestimate the BoS average area and width, while underestimating length, in all tested feet positions. While differences between the Equimetrix BoS and the pressure mat were only found in the FT area and UNI area and width, there is not a clear pattern of reliability and correlation, showing it to be dependent of the feet position. These results are summarized and detailed in Table 8.

The corresponding statistical results and the observed power and effect size can be consulted in Table 9.

### 3.4. Relative Biomechanical Feature Variations

**Figure 7:**
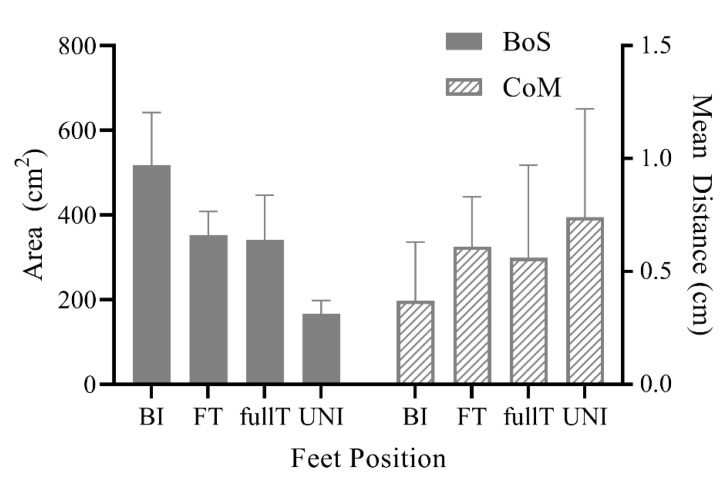
Equimetrix average Base of Support area and Center of mass mean distance during the four postural conditions (BI: bipedal, FT: feet together, UNI: unilateral support over the dominant lower limb and fullT: full tandem with dominant foot forward). BoS data is expressed as mean and standard deviation, while CoM data is presented as median and inter-quartile range.

Due to the intended use of Equimetrix in clinical settings and functional requirements (portable, light weight, and low cost) its two main components (sensing mat and wearable motion tracking module) cannot be as accurate as a force plate/baropodometer and full motion tracking devices, as descried in the previous subsections. It is worth reporting that both the BoS and CoM estimated by the Equimetrix are reflecting intuitive a priori variations. As depicted in Figure 7, BI area is by definition larger than FT area, which is also larger than UNI, while fullT and FT areas are the same. Similarly, the Equimetrix CoM Mean Distance, which correlates to the body’s oscillations, reflects that the system is able to quantify the intuitive a priori variations of stability: BI standing being more stable than FT, which in turn is more stable than fullT, and UNI standing is the least stable.

## 4. Discussion

### 4.1. Center of Mass and Center of Pressure

The identification of differences in the CoM height measured with the K_CoM and *E*_*CoM* was fundamental to allow the comparison of the Equimetrix measurement capability. According to the inverted pendulum model [51] and its trigonometric characteristics, for the same sway angle, a higher CoM vertical component will result in increased absolute AP and ML sway migration. Indeed, Chiari, Rochhi and Cappello [43] reported that several parameters (Mean Velocity, Mean Distance, Range, and RMS) are dependent of biomechanical factors, such as the subject’s height, hence the subject’s CoM height. The need of such a correction step demonstrates the Equimetrix system estimation of the CoM height still needs improvement. This comes from the fact that the internal parameters described in Equation (1) to estimate CoM position based on camera position were constant for all users. A new algorithm version that estimates CoM using personalized information such as the trunk height, waist circumference, camera distance to user’s sacrum, as well as feet position extracted from the sensing mat, is under development and may solve this issue.

However, the fact the Sway Range and Sway RMS value of *E*_*CoM* did not show differences with the K_CoM is an indication that the AP and ML components of the *E*_*CoM* may yet show enough reliability in order to detect similar sway angles. Despite finding differences with the Sway Range and Sway RMS in the BI position, no differences were found in the other tested feet position. It was our expectation that the correction of the *E*_*CoM* height based on the Sway Angle, and therefore of the AP and ML excursion, that the Equimetrix system capabilities to measure these two components could be reliably evaluated.

When analyzing the 1D parameters of the *E*_*CoM*, it is possible to verify that the vertical component obtained from the regression equation does not present any difference in comparison with the K_CoM in terms of RMS value, and that the AP and ML components are identical to those of the K_CoM or the F_CoP. The high reliability and correlation found are a good indicator that, in terms of RMS value, the position of the *E*_*CoM* is comparable to the reference systems. Indeed, the AP value (0.41 (0.29) cm) from the *E*_*CoM* in the BI position is also close to values previously reported [52] for young healthy adults (0.50–1.00 cm).

When analyzing the range of migration, the *E*_*CoM* presents substantially higher values than the K_COM, while keeping similar RMS values. This may indicate that the Equimetrix system has a higher measurement variability, but this is not supported by the corresponding RMS standard deviation and inter-quartile range, further pointing to a possible effect of increased noise in the range results. While differences and low reliability exist for the *E*_*CoM* height measurement during quiet stance, one cannot discard that in more dynamic situations this potential measuring noise can have a smaller effect.

Despite the good spatial measurement of the Equimetrix system, which is in line with the results from the reference systems, the spatial-time accuracy is not as good, as we have initially hypothesized. This can be a result of the short recording time, which Schubert, Kirchner, Schmidtbleicher and Haas [45] have reported to amplify transient effects and is associated to poorer time-resolution. The Path Velocity of *E*_*CoM* differs from the K_CoM in all its components and feet position but is only different from the F_CoP in the BI and fullT at the ML direction. Since the CoP velocity is well correlated with the body’s acceleration [53], this higher *E*_*CoM* velocity may indicate the Equimetrix device is suitable to measure such parameter. However, no measurements were performed in this study to evaluate such a possibility. Despite not showing statistical differences with the F_CoP, the *E*_*CoM* reliability is poor and with non-significant correlations, which is not sufficient to provide reliable and comparable results in terms of velocity.

The 1D components of the CoM and CoP are an important element to determine the body sway in a given direction. However, postural control comprises complex mechanisms that combines sway in both the AP and ML direction in order to keep balance. The statokinesigram allows for a global insight of the CoM and CoP migration over time and can be analyzed by its 2D parameters. During the assessments, the *E*_*CoM* travelled a mean distance identical to the K_CoM and F_CoP in all feet positions, showing an excellent reliability and correlation. Nevertheless, along that distance, the *E*_*CoM* performs a higher travel length than the K_CoM, but not different from that observed for the F_CoP in the FT and fullT positions. The explanation for this difference can be the inherent faster movements of the CoP in order to keep in balance with the CoM position [54]. Still, since the *E*_*CoM* aims at measuring the CoM movement, it should not be subjected to the same characteristics of the CoP. Therefore, the increased length may be related to measurement noise.

The area of the 95% predictive ellipse is similar between systems, thus showing that the simultaneous movement of the *E*_*CoM* along the AP and ML directions produces an overall identical area to the reference systems, which is also associated to an excellent reliability and correlation. The area obtained in the FT position (3.65 (2.39) cm^2^) is also close to the area reported by Harringe, Halvorsen, Renström & Werner [55] (4.26 ± 1.75 cm^2^).

We have initially hypothesized that the Equimetrix would not relate as well with F_CoP parameters as with those originated by the K_CoM. Although that may have been true in some cases, the fact that the *E*_*CoM* did not presented differences with F_CoP in parameters were K_CoM was found to differ, means that our initial assessment was not entirely correct.

Despite the differences and similarities with the reference systems, the *E*_*CoM* presents lower reliability values on the BI position. This is one more evidence that the Equimetrix measuring capacity and reliability is somewhat position-dependent, with more stable positions, such as the BI, showing increased differences with the reference systems, while the more unstable positions present results that are more similar. This was previously observed by Chiari, Rochhi and Cappello [43] and Kim et al. [56], who reported that an increased BoS is reflected as a decrease in ML parameters. This may be due to the fact that in more stable positions, smaller variations in position may be below the system’s associated error. 

### 4.2. Base of Support

Although the BoS measurements were performed in two distinct moments, the use of the feet contours on a sheet of paper enabled the accurate use of the pressure mat. The thickness and damping characteristics of the Equimetrix mat plastic cover, would have caused a diffusion effect of the pressure around the feet. This would not have allowed a precise detection of the feet limits and therefore an unreliable BoS estimate.

Positions with higher base of support areas, such as BI, are associated with lower differences between measuring devices in terms of area and width, but not in length. The base of support area seems to be most influenced by its length component, as this presents the higher amount of feet position with significant differences between measurements devices.

It is important to keep in mind that the Equimetrix sensing elements are larger (2.25 cm^2^) than the ones present in the pressure mat (0.25 cm^2^). This means the data resulting from the pressure mat has nine times more spatial resolution than the Equimetrix, thus making a quite reliable measuring instrument. Taking in consideration the differences in sensor size, it is not surprising the Equimetrix have a tendency to present higher BoS area results, since the smallest touch on a sensor would translate in an increase of 2.25 cm^2^. However, this size difference can also account for the differences between systems. For instance, a statistically significant difference in Equimetrix’ BoS width for the UNI position was observed, which corresponded to an absolute difference between medians of only 0.65 cm^2^, which is a difference smaller than the size of the Equimetrix sensor. 

### 4.3. Study Limitations

The primary limitation of this study was the sample size and gender distribution, as a larger and more homogeneous sample might have provided more robust results. However, the complexity of the experimental setup, and the duration of the tests was an inhibitory factor. Moreover, the fact that the BoS comparison was done sequentially rather than in parallel with the postural stability tests is also a drawback. Since we had to rely on foot contours on a sheet of paper in order to reproduce their positions, we did not entirely control the reproducibility between measuring systems. This method also prevented the investigation of the dynamic changes of the CoM and BoS, such as those occurring during a step. Finally, this study focused on the reliability between Equimetrix and reference systems, not exploring their results agreement, which could have provided more information regarding the possible existence of a bias.

## 5. Conclusions

This was the first validation study on the Equimetrix system, and results support the ability to use it as an alternative in terms of CoM, CoP, and BoS measurement in a quasi-static erect position in humans. The system reported an excellent reliability and correlation in terms of planar (AP and ML) spatial accuracy of the CoM, but caution is advised when interpreting results with a temporal component.

The BoS provided by the Equimetrix has a tendency to be higher than the one reported by a pressure mat, but for the most part similar BoS width and length can be found, enabling their use as limits of stability parameters.

Despite the encouraging results, further improvements are required for a more accurate calculation of the CoM height, since this was proven to be excessive, and could lead to an overestimation of the CoM’s AP and ML movement. Furthermore, validation of dynamic movements is required to verify if the poor performance in some parameters is indeed related to a difficulty in measuring small variation over the measurement noise.

## Figures and Tables

**Table 1:** State of the art technologies suitable for the measurements of biomechanical parameters: Center of Pressure (CoP), Center of Mass (CoM), Base of Support (BoS), and “CoM + BoS” which corresponds to the combination of CoM related measures with BoS information. Static standing: maintaining upright posture. Dynamic standing: feet position fixed while upper body is moving within the limits of stability. Stepping response: during such tasks both upper and lower limbs can move intentionally (planned task) or reactively (response to external perturbations).

	Biomechanical Measurements				Functional Tasks		
	CoP	CoM	BoS	CoM + BoS	Static Standing	Dynamic Standing	Stepping Response
Dynamometric and Stabilometric devices	√				√	√	
Baropodometric device	√		√		√	√	√
Motion capture Systems		√			√	√	√
Inertial measurement units		√			√	√	√
Equimetrix		√	√	√	√	√	√

**Table 2:** Values of the CoM height measured with the kinematic (K_CoM) and Equimetrix (*E*_*CoM*) systems, as well as the Sway Angle Range and Root Mean Square (RMS) value. The CoM Adjusted represents the new *E*_*CoM* height obtained from the regression equation. Parametric data is expressed as Mean ± Standard-deviation, while non-parametric data is express as Median (Inter-quartile range).

Position	Source	CoM Height (cm)	CoM Adjusted (cm)	Sway Angle Range (°)		Sway Angle RMS (°)	
				AP	ML	AP	ML
BI	K_CoM	92.5 (5.9)	-	47.1 ± 11.6	21.8 (9.4)	14.8 (6.4)	5.1 (2.8)
	*E*_*CoM*	117.3 (8.8)	92.9 (5.4)	52.3 (11.5)	34.0 (15.5)	16.2 (6.3)	7.7 (4.2)
FT	K_CoM	92.4 (5.9)	-	49.0 (9.2)	51.7 (8.3)	14.6 (4.6)	15.7 (4.1)
	*E*_*CoM*	117.7 (9.2)	92.2 (5.9)	51.7 (9.2)	54.9 (9.1)	15.5 (5.1)	17.0 (5.1)
fullT	K_CoM	91.2 ± 6.4	-	53.4(14.3)	57.1 (7.7)	20.0 (11.8)	19.4 (7.8)
	*E*_*CoM*	115.8 (9.7)	91.0 (6.8)	56.9 (8.5)	56.9 (8.5)	24.3 (15.9)	19.6 (9.2)
UNI	K_CoM	93.4 ± 5.9	-	60.0 (9.7)	55.0 (7.8)	23.6 (9.7)	17.5 (4.4)
	*E*_*CoM*	118.2 (8.4)	92.6 (5.2)	62.1 (8.2)	59.3 (8.7)	25.1 (9.0)	18.6 (4.9)

Shaded values represent significant differences with *E*_*CoM* for parametric (*p* < 0.05) or non-parametric data (*p* < 0.017). BI: bipodal; FT: feet together; fullT: full tandem; UNI: unipodal.

**Table 3:** Statistic results, power and effect size of the CoM height measured with the kinematic (K_CoM), as well as the Sway Angle Range and Root Mean Square (RMS) value, when compared to the Equimetrix (*E*_*CoM*) measurement. Parametric data is expressed as Mean ± Standard-deviation, while non-parametric data is express as Median (Inter-quartile range).

Position	Statistics	CoM Height	Sway Angle Range		Sway Angle RMS	
			AP	ML	AP	ML
BI	*t*/*U*	4.00	−1.50	101.00	164.00	114.00
	*p*	<0.01	0.14	<0.01	0.34	0.02
	Power	1.00	0.31	0.81	0.16	0.68
	Cohen’s *d*	3.21	0.47	0.95	0.32	0.80
FT	*t*/*U*	3.00	136.00	127.00	147.00	140.00
	*p*	<0.01	0.42	0.28	0.65	0.50
	Power	1.00	0.13	0.19	0.07	0.10
	Cohen’s *d*	3.23	0.28	0.38	0.16	0.24
fullT	*t*/*U*	1.94	120.00	143.00	126.00	132.00
	*p*	<0.01	0.41	0.97	0.54	0.68
	Power	1.00	0.13	0.05	0.09	0.07
	Cohen’s *d*	3.00	0.30	0.02	0.22	0.15
UNI	*t*/*U*	−9.67	107.00	90.00	110.00	109.00
	*p*	<0.01	0.45	0.16	0.52	0.49
	Power	1.00	0.12	0.30	0.10	0.11
	Cohen’s *d*	3.42	0.29	0.53	0.25	0.26

Statistics results from parametric (*t*) or non-parametric tests (*U*) are presented with their significance level (*p*). Shaded values represent significant differences with *E*_*CoM* for parametric (*p* < 0.05) or non-parametric data (*p* < 0.017). NA stands for non-applicable. BI: bipodal; FT: feet together; fullT: full tandem; UNI: unipodal.

**Table 4:** Descriptive and reliability statistical results for the one-dimension parameters for the comparison between Equimetrix CoM (*E*_*CoM*) and the kinematic CoM (K_CoM) and force platform CoP (F_CoP) for the anterior–posterior (AP), medial–lateral (ML) and vertical (V) components. Parametric data is expressed as Mean ± Standard-deviation, while non-parametric data is express as Median (Inter-quartile range).

Position	Source	Mean Path Velocity (cm/s)			Range (cm)			RMS (cm)		
		AP	ML	V	AP	ML	V	AP	ML	V
BI	K_CoM	0.21 (0.10) ^C^	0.1 (0.09) ^D^	0.05 (0.03) ^D^	1.77 (1.12) ^A1^	0.55 (0.70) ^D^	0.24 (0.16) ^D^	0.39 (0.33) ^A1^	0.11 (0.17) ^C1^	92.16 (9.01) ^A1^
	F_CoP	0.32 (0.12) ^C2^	0.2 (0.10) ^D^	NA	1.97 (1.00) ^B1^	0.75 (0.71) ^C1^	NA	0.37 (0.26) ^A1^	0.13 (0.17) ^C2^	NA
	*E*_*CoM*	0.43 (0.14)	0.44 (0.43)	0.46 (0.29)	2.26 (1.65)	0.81 (1.03)	0.9 (0.78)	0.41 (0.29)	0.16 (0.21)	91.93 (6.19)
FT	K_CoM	0.26 (0.10) ^C2^	0.32 (0.14) ^C2^	0.05 (0.02) ^D^	1.81 (1.27) ^B1^	1.94 (0.93) ^A1^	0.26 (0.27) ^C^	0.39 (0.21) ^A1^	0.41 (0.21) ^A1^	91.43 (11.07) ^A1^
	F_CoP	0.35 (0.18) ^C2^	0.5 (0.15) ^C2^	NA	2.04 (0.97) ^B1^	2.22 (1.22) ^B1^	NA	0.39 (0.17) ^A1^	0.44 (0.22) ^A1^	NA
	*E*_*CoM*	0.42 (0.15)	0.57 (0.54)	0.38 (0.11)	2.23 (1.06)	2.32 (1.38)	0.7 (0.29)	0.44 (0.17)	0.48 (0.26)	91.89 (6.13)
fullT	K_CoM	0.25 (0.13) ^C^	0.39 (0.15) ^D2^	0.09 (0.04) ^D^	1.97 (1.92) ^A1^	2.52 ± 0.63^A^	0.63 (0.44) ^D^	0.43 (0.45) ^A1^	0.52 ± 0.17 ^A^	90.4 (9.52) ^B1^
	F_CoP	0.42 (0.22) ^C^	0.63 (0.19) ^C^	NA	2.11 (2.62) ^A1^	2.97 ± 0.89 ^C^	NA	0.43 (0.39) ^A1^	0.56 ± 0.16 ^A^	NA
	*E*_*CoM*	0.42 (0.21)	0.46 (0.23)	0.36 (0.72)	1.95 (1.6)	2.46 ± 0.77	1.12 (1.27)	0.41 (0.40)	0.49 ± 0.17	90.72 (7.23)
UNI	K_CoM	0.46 ± 0.11 ^C3^	0.42 (0.08) ^D^	0.09 (0.07) ^D^	2.57 (2.41) ^A1^	2.01 (0.78) ^B1^	0.78 (0.75) ^C^	0.59 (0.44) ^A1^	0.44 (0.14) ^A1^	92.02 (8.84) ^A1^
	F_CoP	0.78 ± 0.16 ^C4^	0.76 (0.16) ^D^	NA	3.9 (3.44) ^A1^	2.58 (0.93) ^B1^	NA	0.67 (0.45) ^A1^	0.49 (0.16) ^A1^	NA
	*E*_*CoM*	0.66 ± 0.23	0.56 (0.32)	0.45 (0.31)	2.85 (2.26)	2.33 (1.33)	1.74 (1.15)	0.65 (0.40)	0.48 (0.24)	92.85 (6.80)

Upper case letters represent the ICC result as excellent (A: ≥0.90), good (B: 0.80–0.89), fair (C: 0.70–0.79) or poor (D: ≤0.69), respectively. Numbers between 1 and 4 represent the Pearson or Spearman correlation as strong (1: 0.75–1.0), moderate (2: 0.50–0.74), poor (3: 0.25–0.49) and little or none (4: 0.00–0.24), respectively. Only Spearman correlations with *p* < 0.01 are presented. Shaded values represent significant differences with *E*_*CoM* for parametric (*p* < 0.05) or non-parametric data (*p* < 0.017). NA stands for non-applicable. BI: bipodal; FT: feet together; fullT: full tandem; UNI: unipodal.

**Table 5:** Statistical results, power, and effect size for the comparison between Equimetrix CoM (*E*_*CoM*) and the kinematic CoM (K_CoM) and force platform CoP (F_CoP) for the anterior–posterior (AP), medial–lateral (ML) and vertical (V) components of the one-dimension parameters.

Position	Comparison	Statistics	Mean Path Velocity			Range			RMS		
			AP	ML	V	AP	ML	V	AP	ML	V
BI	K_CoM vs. *E*_*CoM*	*t*/*U*	22.00	14.00	0.00	114.00	33.00	24.00	150.00	104.00	179.00
		*p*	<0.01	<0.01	<0.01	0.05	0.01	<0.01	0.39	0.03	0.98
		Power	1.00	1.00	1.00	0.57	0.83	1.00	0.15	0.70	0.05
		Cohen’s *d*	2.34	2.66	3.47	0.67	0.93	2.28	0.24	0.79	0.01
	F_CoP vs. *E*_*CoM*	*t*/*U*	65.00	76.00	NA	134.00	131.00	NA	149.00	129.00	NA
		*p*	<0.01	<0.01		0.18	0.15		0.37	0.14	
		Power	0.99	0.96		0.30	0.34		0.16	0.36	
		Cohen’s *d*	1.33	1.16		0.46	0.49		0.31	0.51	
FT	K_CoM vs. *E*_*CoM*	*t*/*U*	36.00	75.00	0.00	143.00	135.00	23.00	162.00	152.00	173.00
		*p*	<0.01	<0.01	<0.01	0.28	0.19	<0.01	0.60	0.42	0.84
		Power	1.00	0.96	1.00	0.21	0.29	1.00	0.09	0.14	0.06
		Cohen’s *d*	1.93	1.17	3.47	0.37	0.45	2.31	0.18	0.28	0.07
	F_CoP vs. *E*_*CoM*	*t*/*U*	123.00	150.00	NA	169.00	178.00	NA	169.00	180.00	NA
		*p*	0.10	0.39		0.75	0.95		0.75	1.00	
		Power	0.44	0.15		0.06	0.05		0.06	0.05	
		Cohen’s *d*	0.57	0.30		0.11	0.02		0.11	0.01	
fullT	K_CoM vs. *E*_*CoM*	*t*/*U*	30.00	60.00	1.00	85.00	NA	37.00	94.00	NA	98.00
		*p*	<0.01	0.09	<0.01	0.57	1.00	<0.01	0.87	1.00	1.00
		Power	0.98	0.48	1.00	0.10	0.08	0.93	0.05	0.08	0.05
		Cohen’s *d*	1.51	0.71	3.34	0.23	0.21	1.28	0.07	0.20	0.00
	F_CoP vs. *E*_*CoM*	*t*/*U*	91.00	44.00	NA	84.00	NA	NA	95.00	NA	NA
		*p*	0.77	0.01		0.54	0.13		0.91	0.78	
		Power	0.06	0.83		0.10	0.46		0.05	0.20	
		Cohen’s *d*	0.12	1.09		0.25	0.79		0.05	0.47	
UNI	K_CoM vs. *E*_*CoM*	*t*/*U*	NA	32.00	2.00	83.00	66.00	31.00	92.00	81.00	92.00
		*p*	0.04	0.01	0.01	0.51	0.15	<0.01	0.80	0.45	0.80
		Power	0.67	0.97	1.00	0.11	0.35	0.98	0.060	0.13	0.06
		Cohen’s *d*	−1.03	1.44	3.21	0.27	0.59	1.47	0.11	0.30	0.11
	F_CoP vs. *E*_*CoM*	*t*/*U*	NA	47.00	NA	78.00	86.00	NA	90.00	86.00	NA
		*p*	0.39	0.02		0.38	0.60		0.73	0.60	
		Power	0.26	0.77		0.16	0.09		0.07	0.09	
		Cohen’s *d*	0.56	1.01		0.36	0.21		0.14	0.21	

Statistics results from parametric (*t*) or non-parametric tests (*U*) are presented with their significance level (*p*). Shaded values represent significant differences with *E*_*CoM* for parametric (*p* < 0.05) or non-parametric data (*p* < 0.017). NA stands for non-applicable. BI: bipodal; FT: feet together; fullT: full tandem; UNI: unipodal.

**Table 6:** Descriptive and reliability statistical results of the two-dimension parameters for the Equimetrix CoM (*E*_*CoM*) comparison with the kinematic CoM (K_CoM) and force platform CoP (F_CoP). Parametric data is expressed as Mean ± Standard-deviation, while non-parametric data is express as Median (Inter-quartile range).

Position	Source	Mean Distance (cm)	Length (cm)	Ellipse Area (cm^2^)	Ellipse Long Axis (cm)	Ellipse Short Axis (cm)
BI	K_CoM	0.35 (0.24) ^A1^	12.57 (6.66) ^C^	0.86 (1.08) ^C1^	0.95 (0.79) ^A1^	0.27 (0.25) ^C^
	F_CoP	0.35 (0.25) ^A^	19.36 (6.63) ^C^	1.11 (1.35) ^C1^	0.90 (0.62) ^A1^	0.32 (0.34) ^C2^
	*E*_*CoM*	0.37 (0.26)	33.10 (27.11)	1.34 (2.67)	1.00 (0.71)	0.39 (0.46)
FT	K_CoM	0.53 (0.21) ^A1^	20.13 (8.32) ^C2^	3.03 (2.00) ^A1^	1.16 (0.56) ^A1^	0.78 (0.28) ^A1^
	F_CoP	0.56 (0.17) ^A1^	30.58 (10.76) ^C2^	3.38 (2.00) ^A1^	1.20 (0.50) ^A1^	0.82 (0.28) ^A1^
	*E*_*CoM*	0.59 (0.20)	39.91 (23.07)	3.65 (2.39)	1.29 (0.50)	0.82 (0.36)
fullT	K_CoM	0.58 (0.46) ^A1^	23.15 (10.25) ^D^	3.44 (6.99) ^A1^	1.45 (0.99) ^A1^	0.79 (0.8) ^A1^
	F_CoP	0.58 (0.40) ^A1^	37.27 (13.88) ^C^	4.17 (6.27) ^A1^	1.47 (0.96) ^A1^	0.96 (0.56) ^A2^
	*E*_*CoM*	0.56 (0.41)	32.97 (14.89)	3.55 (5.35)	1.39 (0.94)	0.79 (0.58)
UNI	K_CoM	0.66 (0.48) ^A1^	27.53 (6.78) ^D^	4.92 (7.72) ^A1^	1.49 (1.07) ^A1^	0.95 (0.34) ^A1^
	F_CoP	0.73 (0.47) ^A1^	50.61 (11.27) ^C^	6.66 (7.59) ^A1^	1.70 (1.05) ^A1^	1.12 (0.36) ^A1^
	*E*_*CoM*	0.74 (0.48)	35.86 (9.56)	5.75 (8.06)	1.68 (0.95)	1.06 (0.51)

Upper case letters represent the ICC result as excellent (A: ≥0.90), good (B: 0.80–0.89), fair (C: 0.70–0.79) or poor (D: ≤0.69), respectively. Numbers between 1 and 4 represent the Pearson or Spearman correlation as strong (1: 0.75–1.0), moderate (2: 0.50–0.74), poor (3: 0.25–0.49) and little or none (4: 0.00–0.24), respectively. Only Spearman correlations with *p* < 0.01 are presented. Shaded values represent significant differences with *E*_*CoM* for parametric (*p* < 0.05) or non-parametric data (*p* < 0.017). BI: bipodal; FT: feet together; fullT: full tandem; UNI: unipodal.

**Table 7:** Statistics results, power, and effect size for the comparison between Equimetrix CoM (*E*_*CoM*) and the kinematic CoM (K_CoM) and force platform CoP (F_CoP) for two-dimension parameters.

Position	Comparison	Statistics	Mean Distance	Length	Ellipse Area	Ellipse Long Axis	Ellipse Short Axis
BI	K_CoM vs. *E*_*CoM*	*t*/*U*	136.00	11.00	120.00	145.00	106.00
		*p*	0.20	<0.01	0.08	0.31	0.03
		Power	0.28	1.00	0.48	0.19	0.67
		Cohen’s *d*	0.44	2.80	0.61	0.35	0.77
	F_CoP vs. *E*_*CoM*	*t*/*U*	147.00	59.00	143.00	145.00	133.00
		*p*	0.34	<0.01	0.28	0.31	0.17
		Power	0.18	0.99	0.21	0.19	0.31
		Cohen’s *d*	0.33	1.44	0.37	0.35	0.47
FT	K_CoM vs. *E*_*CoM*	*t*/*U*	149.00	43.00	148.00	146.00	161.00
		*p*	0.37	<0.01	0.35	0.33	0.58
		Power	0.16	1.00	0.17	0.19	0.09
		Cohen’s *d*	0.31	1.76	0.32	0.37	0.19
	F_CoP vs. *E*_*CoM*	*t*/*U*	166.00	132.0	172.00	167.00	166.00
		*p*	0.69	0.16	0.82	0.71	0.69
		Power	0.07	0.33	39.67	0.07	0.07
		Cohen’s *d*	0.14	0.48	0.08	0.13	0.14
fullT	K_CoM vs. *E*_*CoM*	*t*/*U*	36.00	34.00	95.00	98.00	97.0
		*p*	0.35	<0.01	0.91	1.00	0.98
		Power	0.05	0.96	0.05	0.05	0.05
		Cohen’s *d*	0.04	1.37	0.05	0.000	0.02
	F_CoP vs. *E*_*CoM*	*t*/*U*	86.00	63.00	85.0	87.00	79.00
		*p*	0.60	0.11	0.57	0.64	0.40
		Power	0.07	0.09	0.41	0.10	0.08
		Cohen’s *d*	0.16	0.21	0.65	0.23	0.20
UNI	K_CoM vs. *E*_*CoM*	*t*/*U*	91.00	20.00	81.00	94.00	78.00
		*p*	0.77	<0.01	0.45	0.87	0.38
		Power	0.06	1.00	0.13	0.05	0.16
		Cohen’s *d*	0.12	1.91	0.30	0.07	0.360
	F_CoP vs. *E*_*CoM*	*t*/*U*	87.00	42.00	88.0	92.00	88.00
		*p*	0.64	<0.01	0.67	0.80	0.67
		Power	0.15	0.08	0.86	0.08	0.06
		Cohen’s *d*	0.34	0.20	1.14	0.18	0.11

Statistics results from parametric (*t*) or non-parametric tests (*U*) are presented with their significance level (*p*). NA stands for non-applicable. Shaded values represent significant differences with *E*_*CoM* for parametric (*p* < 0.05) or non-parametric data (*p* < 0.017). BI: bipodal; FT: feet together; fullT: full tandem; UNI: unipodal.

**Table 8:** Descriptive and reliability statistical results of the base of support parameters between the Equimetrix device and the pressure mat. Parametric data is expressed as Mean ± Standard-deviation, while non-parametric data is express as Median (Inter-quartile range).

Feet Position	Pressure Mat			Equimetrix		
	Average Area (cm^2^)	Width (cm)	Length (cm)	Average Area (cm^2^)	Width (cm)	Length (cm)
BI	502.1 ± 78.58 ^C2^	26.36 ± 2.32 ^A1^	22.6 (2.5) ^B2^	517.65 ± 124.32	26.97 ± 3	21.35 (5.1)
FT	287.4 ± 48.01 ^A1^	16.8 (2.3) ^B1^	21.88 ± 2.49 ^C2^	352.85 ± 55.09	17.5 (2.6)	21.5 ± 1.78
fullT	305.1 ± 60.71 ^C2^	9.2 (1.7) ^B2^	46.1 (6.6) ^C1^	340.85 ± 105.99	9.35 (2.4)	42.65 (7.4)
UNI	117.7 ± 22.01 ^B1^	7.5 (1.7) ^D3^	22.17 ± 1.87 ^B1^	166.05 ± 32.31	8.15 (2.4)	21.92 ± 1.74

Upper case letters represent the ICC result as excellent (A: ≥0.90), good (B: 0.80–0.89), fair (C: 0.70–0.79) or poor (D: ≤0.69), respectively. Numbers between 1 and 4 represent the Pearson or Spearman correlation as strong (1: 0.75–1.0), moderate (2: 0.50–0.74), poor (3: 0.25–0.49) and little or none (4: 0.00–0.24), respectively. Only Spearman correlations with *p* < 0.01 are presented. Shaded values represent significant differences with *E*_*CoM* for parametric (*p* < 0.05) or non-parametric data (*p* < 0.017). BI: bipodal; FT: feet together; fullT: full tandem; UNI: unipodal.

**Table 9:** Statistical results, power, and effect size of the base of support parameters between the Equimetrix device and the pressure mat.

Feet Position	Average Area			Width				Length		
	*t*/*U* (*p*)	Power	Cohen’s *d*		*t*/*U* (*p*)	Power	Cohen’s *d*	*t*/*U* (*p*)	Power	Cohen’s *d*
BI	0.47 (0.64)	0.15	0.05		0.71 (0.48)	0.23	0.11	133.50 (0.07)	0.60	0.50
FT	4.01 (<0.01)	1.27	0.97		149.00 (0.17)	0.46	0.31	−0.56 (0.58)	−0.18	0.09
fullT	1.31 (0.20)	0.41	0.22		188.00 (0.76)	0.11	0.06	133.00 (0.07)	0.61	0.50
UNI	5.53 (<0.01)	1.75	1.00		99.50 (<0.01)	0.98	0.89	−0.45 (0.66)	−0.14	0.07

Statistics results from parametric (*t*) or non-parametric tests (*U*) are presented with their significance level (*p*). Shaded values represent significant differences with *E*_*CoM* for parametric (*p* < 0.05) or non-parametric data (*p* < 0.017). BI: bipodal; FT: feet together; fullT: full tandem; UNI: unipodal.

## Data Availability

The data presented in this study are available on request from the corresponding author. The data are not publicly available due to on-going development.
